# An RNA-Seq based gene expression atlas of the common bean

**DOI:** 10.1186/1471-2164-15-866

**Published:** 2014-10-06

**Authors:** Jamie A O’Rourke, Luis P Iniguez, Fengli Fu, Bruna Bucciarelli, Susan S Miller, Scott A Jackson, Philip E McClean, Jun Li, Xinbin Dai, Patrick X Zhao, Georgina Hernandez, Carroll P Vance

**Affiliations:** Department of Agronomy and Plant Genetics, University of Minnesota, St. Paul, MN 55108 USA; Centro de Ciencias Genomicas, Universidad Nacional Autonoma de Mexico, 66210 Cuernavaca, Mor Mexico; USDA-Agricultural Research Service, Plant Science Research Unit, St. Paul, MN 55108 USA; Center for Applied Genetic Technologies, University of Georgia, Athens, GA 30602 USA; Department of Plant Sciences, North Dakota State University, Fargo, ND 58105 USA; Plant Biology Division, The Samuel Roberts Noble Foundation, Ardmore, OK 73401 USA; USDA-ARS, Corn Insect Crop Genetics Research Unit, Iowa State University, Ames, IA 50011 USA

**Keywords:** *Phaseolus vulgaris* cv Negro jamapa, Common bean, RNA-Seq, Symbiotic nitrogen fixation, Expression atlas, SRP046307

## Abstract

**Background:**

Common bean (*Phaseolus vulgaris*) is grown throughout the world and comprises roughly 50% of the grain legumes consumed worldwide. Despite this, genetic resources for common beans have been lacking. Next generation sequencing, has facilitated our investigation of the gene expression profiles associated with biologically important traits in common bean. An increased understanding of gene expression in common bean will improve our understanding of gene expression patterns in other legume species.

**Results:**

Combining recently developed genomic resources for *Phaseolus vulgaris,* including predicted gene calls, with RNA-Seq technology, we measured the gene expression patterns from 24 samples collected from seven tissues at developmentally important stages and from three nitrogen treatments. Gene expression patterns throughout the plant were analyzed to better understand changes due to nodulation, seed development, and nitrogen utilization. We have identified 11,010 genes differentially expressed with a fold change ≥ 2 and a P-value < 0.05 between different tissues at the same time point, 15,752 genes differentially expressed within a tissue due to changes in development, and 2,315 genes expressed only in a single tissue. These analyses identified 2,970 genes with expression patterns that appear to be directly dependent on the source of available nitrogen. Finally, we have assembled this data in a publicly available database, The *Phaseolus vulgaris* Gene Expression Atlas (*Pv* GEA), http://plantgrn.noble.org/PvGEA/ . Using the website, researchers can query gene expression profiles of their gene of interest, search for genes expressed in different tissues, or download the dataset in a tabular form.

**Conclusions:**

These data provide the basis for a gene expression atlas, which will facilitate functional genomic studies in common bean. Analysis of this dataset has identified genes important in regulating seed composition and has increased our understanding of nodulation and impact of the nitrogen source on assimilation and distribution throughout the plant.

**Electronic supplementary material:**

The online version of this article (doi:10.1186/1471-2164-15-866) contains supplementary material, which is available to authorized users.

## Background

Common bean (*Phaseolus vulgaris* L.), *Pv,* is an important source of proteins, micronutrients and calories for over three hundred million people worldwide, mostly throughout Latin America and Africa where beans are an important component of traditional diets. The high levels of dietary protein (between 20 and 25%) and micronutrients in beans complement the high carbohydrates found in maize and cassava [1]. In addition to their important contribution to human health, legumes are also important contributors to biological nitrogen (N). N is a primary nutrient limiting plant production [2], with the acquisition and assimilation of N second only to photosynthesis for plant growth and development [1]. Despite the international importance of *Pv*, both in terms of economics and consumption, it has lagged behind *Medicago truncatula, Lotus japonicus,* soybean, and other legumes in terms of genetic resources. cDNA libraries have been used to investigate phosphate stress, resistance to bean rust, and leaf development [3–7]. Sequence information for *Pv* was greatly enhanced by using Roche 454 technology coupled with mRNA sequences to assemble 59,295 unigene sequences, [8], though these data are not yet publicly available. Most recently, the genome sequence and predicted gene calls for *Pv* G 19833 has been made publicly available (http://www.phytozome.net). This resource provides a platform for *Pv* genomic and comparative genomic analyses [9]. Sequence conservation and genetic colinearity between *Pv* and soybean (*Glycine max* L. merr) [10, 11] which diverged from a common ancestor approximately 19 million years ago [12, 13], allows genomic information to be leveraged from one species to the other.

In this study we utilized RNA-seq to characterize expression profiles for the transcriptome of common bean (*Pv* cv. Negro Jamapa). Gene expression profiles were analyzed from 24 unique samples from seven distinct tissues; roots, nodules, stems, flowers, leaves, pods, and seeds throughout development. Our data was used as the foundation for The *Pv* Gene Expression Atlas (*Pv* GEA) database, available at http://plantgrn.noble.org/PvGEA/. We utilized the expression profiles of all predicted genes in *Pv* to examine the biological processes related to seed and pod development, nodulation and symbiosis, and changes in gene expression due to nitrogen availability.

## Results and discussion

### *Phaseolus vulgaris*gene expression atlas (*Pv*GEA)

To facilitate additional use of the RNA-Seq data generated for these analyses, we have developed a web-accessible database, The *Pv* Gene Expression Atlas (*Pv* GEA), available at http://plantgrn.noble.org/PvGEA/. This database was built using a similar database structure, web application, architecture and tools as the LegumeIP platform [14] to retrieve and visualize the gene expression patterns using RNA-seq data. To facilitate the mining of the data included in *Pv* GEA, we have provided the capability to: (i) visualize expression profiles of genes of interest, (ii) identify genes exhibiting certain expression patterns in specific tissues, (iii) identify genes and gene expression patterns based on http://www.phytozome.net annotation terms; and (iv) download the entire data set, either raw or normalized, in tabular form to facilitate the analysis of more complicated biological questions. Using the predicted gene calls of the G 19833 *Pv* genome to build the *Pv* GEA database means it can be easily expanded to integrate RNA-Seq data from future experiments. Currently, *Pv* GEA includes gene expression profiles from 24 samples isolated from roots, root nodules, stems, leaves, flowers, pods, and seeds at various developmental stages under ideal growth conditions. Included in this dataset are transcripts from eight samples including nodule, root, and leaf tissues for plants having either fix + or fix- root nodules; providing preliminary data on the impact of nodulation and N fixation on gene expression, an important biological process for legumes.

The 26,964 transcriptionally active genes identified in our data (RPKM ≥ 3 in at least one tissue) represent 78% of the 31,638 predicted genes in *Pv*; confirming that the tissues, time points, and treatments used in this study (as described in Table 1) affected a majority of the genes in the genome and provide an excellent foundation of gene expression in future experimental comparisons. Pair-wise analyses identified differentially expressed genes between both tissues and samples (Table 2 and Additional file 1, respectively). These comparisons identified 11,010 genes differentially expressed between tissues. Additionally, we identified genes differentially expressed within tissues from samples collected at developmentally important time-points (between seeds from three developmental stages (DS): 2,694, four pod DS: 13,125, five leaf DS: 5,401, six root DS: 1,458, and three nodule DS: 1,551). Finally, we identified genes exhibiting either tissue specific expression, (Additional file 2 and Additional file 3a), or sample specific expression, (Additional file 3b and Additional file 4).

**Table 1:** **Tissue samples isolated from** ***Phaseolus vulgaris*** **cv. Negro jamapa for RNA-Seq analysis**

Organ	Sample ID ^a^	Sample description	Reads sequenced ^b^	Reads mapped ^c^	Genes expressed ^d^
Leaves	YL	Fully expanded 2^nd^ trifoliate leaf tissue from plants provided with fertilizer	24,118,479	21,395,546	19,466
	L5	Leaf tissue collected 5 DAI with effective rhizobium	25,297,092	19,762,749	18,966
	LF	Leaf tissue from fertilized plants collected at the same time of LE and LI	22,692,275	17,581,928	16,103
	LE	Leaf tissue collected 21 DAI with effective *rhizobium*	23,366,279	17,714,097	16,081
	LI	Leaf tissue collected 21 DAI with ineffective *rhizobium*	23,257,968	18,820,812	18,523
Stem	YS	All stem internodes above the cotyledon collected at the 2^nd^ trifoliate stage	27,696,970	22,718,724	20,299
	ST	Shoot tip, including the apical meristem, collected at the 2^nd^ trifoliate stage	25,826,838	22,358,410	21,142
Flower	FY	Young flowers, collected prior to floral emergence	23,334,037	16,503,930	20,055
Pods	PY	Young pods, collected 1 to 4 days after floral senescence. Samples contain developing embryos at globular stage	26,234,498	17,381,695	12,115
	PH	Pods approximately 9 cm long, associated with seeds at heart stage (pod only)	24,986,174	19,130,051	19,065
	P1	Pods between 10 and 11 cm long, associated with stage 1 seeds (pod only)	24,349,622	16,400,591	15,831
	P2	Pods between 12 and 13 cm long associated with stage 2 seeds (pod only)	21,647,774	18,018,224	18,311
Seeds	SH	Heart stage seeds, between 3 and 4 mm across and ~7 mg	28,222,798	22,972,385	18,668
	S1	Stage 1 seeds, between 6 and 7 mm across and ~50 mg	21,395,296	17,004,466	16,949
	S2	Stage 2 seeds, between 8 and 10 mm across and 140–150 mg	24,696,630	19,949,204	15,363
Roots	RT	Root tips, 0.5 cm of tissue, collected from fertilized plants at 2^nd^ trifoliate stage of development	24,536,948	21,680,391	18,514
	YR	Whole roots, including root tips, collected at the 2^nd^ trifoliate stage of development	25,140,904	20,962,342	19,170
	R5	Whole roots separated from 5 day old pre-fixing nodules	27,423,246	23,034,248	19,865
	RF	Whole roots from fertilized plants collected at the same time as RE and RI	25,121,968	21,858951	20,305
	RE	Whole roots separated from fix + nodules collected 21 DAI	24,449,104	21,325,105	20,450
	RI	Whole roots separated from fix- nodules collected 21 DAI	27,834,770	24,188,904	20,697
Nodules	N5	Pre-fixing (fix+) nodules collected 5 DAI	23,909,973	20,774,095	19,102
	NE	Fix + nodules collected 21 DAI	26,350,845	23,035,381	17,011
	NI	Fix- nodules collected 21 DAI	24,875,317	21,877,973	19,278

^a^A two-letter ID assigned to each sample. ^b^Number of single end 36 bp reads generated for each sample. ^c^Number of RNA-Seq reads mapping to the genome using Bowtie. ^d^Number of predicted genes expressed with an RPKM ≥ 3 in each sample.

**Table 2:** **Differentially expressed genes between tissue types**

	Seed	Pod	Stem	Leaf	Root	Nodule
Seed	0	909	2,521	446	1,690	840
Pod	665	0	227	844	504	455
Stem	1,299	946	0	1,847	602	773
Leaf	1,354	1,111	690	0	1,112	1,084
Root	1,986	1,867	731	3,003	0	679
Nodule	1,554	936	526	1,789	375	0

The number of genes in each cell represents genes up regulated in the column tissue compared to the row tissue. For the comparison Seeds vs Pods 1,574 genes are differentially expressed; 909 up regulated in pods and 665 up-regulated in seeds.

To ensure our transcriptome analysis was reliable we visualized the expression profile of the purine and ureide biosynthesis pathways (Additional file 5) and conducted qPCR on 85 additional genes (for details see experimental procedures and Additional file 6). In warm season legumes, such as soybean and *Pv*, purine biosynthesis is known to be highly up regulated in nodules [15]. Our data is consistent with this as the genes in this pathway are highly up regulated in nitrogen fixing nodules compared to all other tissues (Additional file 5). The enzyme uricase (Additional file 3c) degrades ureate to allantoin, which is the main supply of N for plant nutrition. In our *Pv* data allantoinase, the enzyme responsible for allantoin degradation, is highly expressed early in seed and pod development, likely providing N to developing seeds (Additional file 3d). Expression of uricase and allantoinase in aerial tissues suggests ureides are degraded after being transported from the nodules. Leaves, seeds, and pods can then utilize the released NH_3_ and CO_2_ in a variety of cellular processes. These results are consistent with reports of high ureide levels observed in developing *Pv* seeds [16] and high allantoinase enzyme activity throughout pod development and seed filling as measured by Thomas *et al*. [17]. Additionally, Pellissier et al. [18] reported allantoin transporters highly expressed in developing pods and seed coats of *Pv*. The results of these three studies combined with the gene expression patterns observed in our data highlight the importance of ureide metabolism in aerial tissues to provide N for developing tissues.

### Gene expression analysis

Genes exhibiting tissue specific expression (Additional file 2 and Additional file 3a) are involved in a variety of gene ontology biological processes. Genes uniquely expressed in leaves are involved in amino acid phosphorylation, DNA and protein binding. Genes uniquely expressed in seed tissues include processes such as carbohydrate metabolism, as exemplified by a starch branching enzyme (SBE), which is important in amylopectin synthesis, a carbohydrate precursor [19]. In our data, *Pv SBE* expression (Phvul.005G040300.1) is highest in developing seeds (Additional file 3e). These results are consistent with the high carbohydrate composition of *Pv* seeds reported by Broughton et al. [1]. Nodule specific transcripts annotate as involved in oxidoreductase activity, amino acid phosphorylation and membrane transport/signaling are highly and uniquely expressed; reflecting the importance of nutrient transport to the nodule and the high energy cost of N fixation. In root tissues, genes involved in pectinecterase, carbohydrate metabolism, iron ion binding, oligopeptide membrane transport, and lipid metabolisms are uniquely expressed. Expression of these genes illustrates the role of the root in nutrient acquisition and the importance of root growth for plant health.

**Figure 1:**
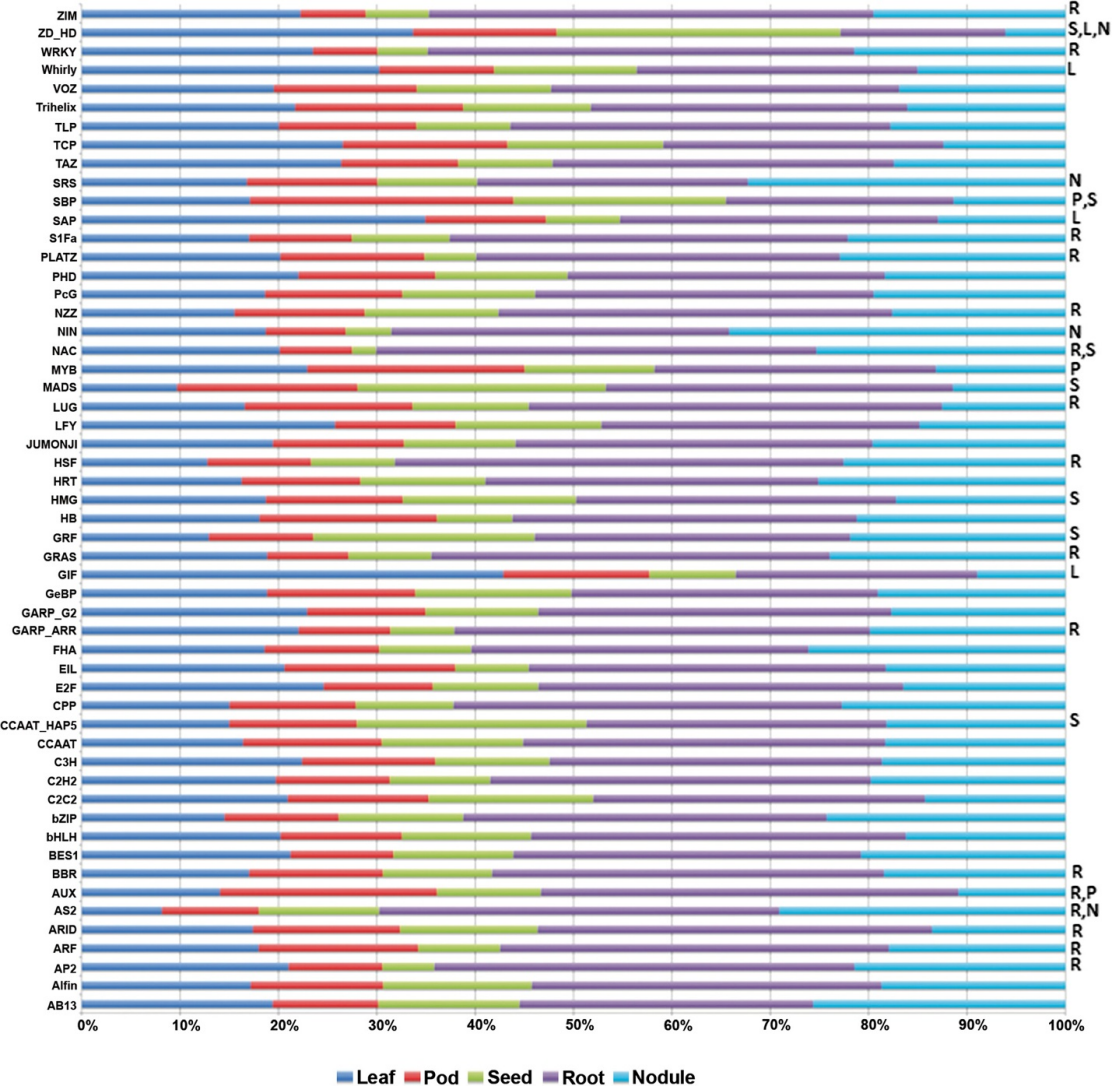
**Transcription factor family expression profile by tissue.** Fisher’s test identified 26 transcription factor families with higher or lower than expected expression in a specific tissue (leaf, blue; pod, red; seed, green; root, purple; nodule, teal). Tissues with statistically significant gene expression patterns are denoted to the right of the graph; Leaf, L; Pod, P; Seed, S; Root, R; Nodule, N.

The 6,667 known transcription factors (TF) in soybean (downloaded from SoyDB) [20] were compared to *Pv* genes using TBLASTN (e-value 1e-30). This analysis identified 3,726 putative TFs in the *Pv* genome, representing 52 of the 64 transcription factor families in soybean (Additional file 7). The 3,726 TFs identified in *Pv* is almost exactly half the number identified in soybean, as expected since soybean has undergone a whole genome duplication event not experienced by *Pv* [12, 13]. The average expression of TFs in all seed tissues was much lower than that of other tissues, including developing pods. Fisher tests confirmed 26 TF families exhibited statistically enhanced or repressed expression by tissue type (Figure 1). Twenty-one TF families exhibited altered expression patterns in a single tissue. Five other TF families (AS2, AUX, NAC, SBP, and ZD-HD) showed expression patterns that were statistically significant in multiple tissues.

### Seed development and metabolism

**Figure 2:**
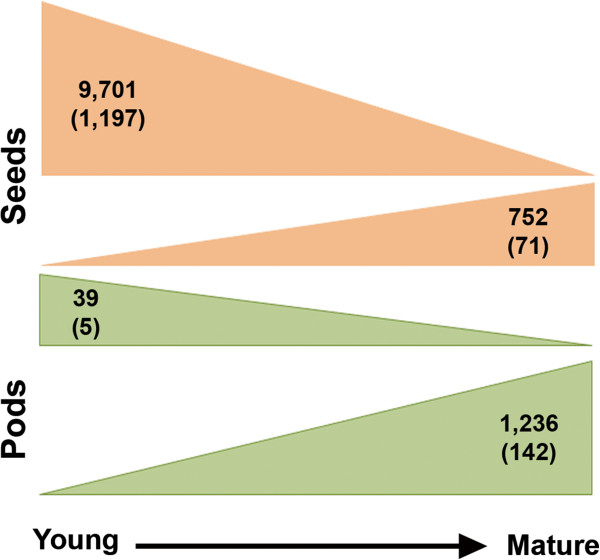
**Expression trends in seed and pod development.** Genes with consistent expression patterns as seeds and pods develop, transcription factors denoted in parentheses.

*Pv* seeds are an integral component of diets around the world. Unlike soybean seeds, which are valued for high oil and protein content, *Pv* beans provide high levels of protein and carbohydrates making it a highly nutritious food for human consumption. The seed and pod samples represent an extended time-course collection of the same tissue spanning great developmental changes. Approximately half (16,292) of the 31,638 predicted genes are expressed in *Pv* seeds with 12,182 genes expressed in all three stages of seed development examined. In *Pv* pods 17,248 genes are expressed in at least one of the four developmental stages examined. We identified genes differentially expressed between seeds and pods at the three stages of development (8,189 genes; Additional file 8, Additional file 9, and Additional file 10), and genes differentially expressed within seeds (2,694) and pods (13,125) throughout development (Additional file 11 and Additional file 12). Additionally, we identified 9,702 genes with consistently decreasing expression levels as the seed develops, including 1,196 TFs. By comparison, 753 genes were identified with increasing expression levels as the seed develops (Figure 2) including 70 TFs from 25 families, including HB (Phvul.007G064100.1), MYB (Phvul.001G025200.1), and ARF (Phvul.011G080100.1) (Additional file 3f). Members of three of these TF families (HB, MYB, and ARF) are seed specific in Arabidopsis, *M. truncatula*, and our *Pv* data [21, 22]. Additionally, members of these TF families are among the TFs identified by Le *et al.* [23] as differentially expressed between structures of developing *Phaseolus coccineus* (scarlet runner bean) seeds that are also differentially expressed in *Pv* seeds at different developmental stages. All these results are consistent with a study by Hajduch *et al*. [24], which determined the expression of proteins involved in primary and secondary metabolism, cell growth and division, signal transduction, and protein synthesis all decrease as the seeds develop. Conversely, in the pods we identified 39 genes with expression levels steadily decreasing and 1,236 genes with consistently increasing expression patterns (Figure 2).

**Figure 3:**
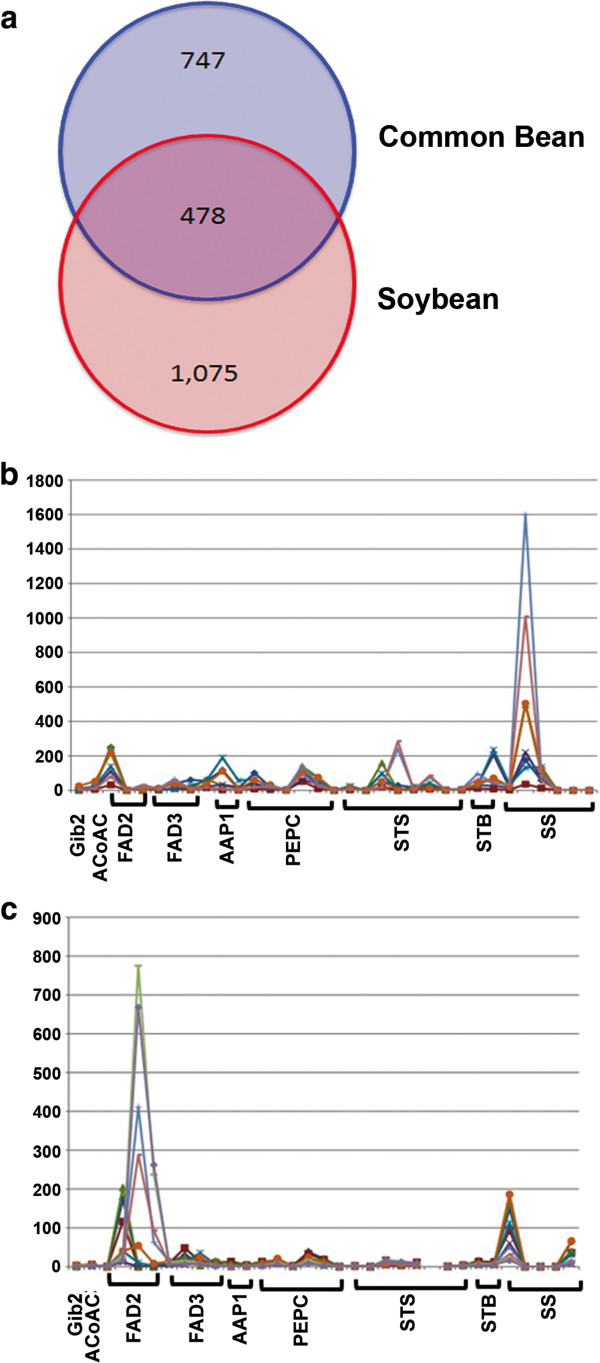
**Comparison of soybean and common bean seeds. (a)** Comparing the top 1,500 expressed genes (regardless of seed stage) in soybean (as reported by Severin *et al*. [26]) and common bean seeds. **(b and c)** Expression profiles of genes involved in fatty acid and starch biosynthesis pathways in developing seed tissues. Glb2 (GLABARA 2), ACoAC (Acetyl CoA Carboxylase), FAD2 and FAD3 (fatty acid desaturase), AAP1 and 2 (amino acid transporter), PEPC (phosphoenolpyruvate carboxylase), STS (starch synthase), STB (starch branching enzyme), SS (sucrose synthase). **(b)** Gene expression profiles in common bean, **(c)** Gene expression profiles in soybean (as reported by Severin *et al*. [26]).

Soybean and *Pv*, although closely related species, have distinct seed compositions. While soybean accumulates oil and protein, *Pv* accumulates carbohydrates and protein [1, 25]. Comparing the 500 most highly expressed genes in both *Pv* and soybean seeds [26] allowed us to identify genes important for general seed development (Figure 3a). Genes involved in carbohydrate biosynthesis are highly up regulated in *Pv* seeds while genes involved in fatty acid biosynthesis are highly up regulated in developing soybean seeds (Figure 3b and c). The starch synthase (STS) genes, particularly Phvul.001G082500.1, are highly expressed in *Pv* seeds (RPKMs: S1 = 240, S2 = 286) but not in soybean seeds (RPKM < 15) (Figure 3b and c). STS is required for amylopectin biosynthesis, a component of carbohydrates. Similarly, sucrose acts as a key regulator of seed carbon flux; high sucrose synthase (SS) activity in developing seeds may channel available carbon towards carbohydrate biosynthesis and away from fatty acid biosynthesis [27]. In our *Pv* RNA-Seq data, genes encoding SS are highly expressed in developing seeds (Figure 3b). In Arabidopsis, SS loss of function mutants favor fatty acid and protein biosynthesis over starch biosynthesis in seed development, resulting in a 55% increase of fatty acids and a near 70% reduction in starch content of mature seeds [28]. The role of SS in regulating carbohydrate synthesis is further supported by the low expression in developing soybean seeds (Figure 3c) (Severin et al. [26]), which are valued for oil and protein. The synthesis of polyunsaturated fatty acids is regulated by FATTY ACID DESATURASE 2 (FAD2) [29]. In soybeans, *FAD2* is highly expressed in developing seeds while in *Pv FAD2* is expressed early in seed and pod development, but at a much lower level (Figure 3b and c).

**Figure 4:**
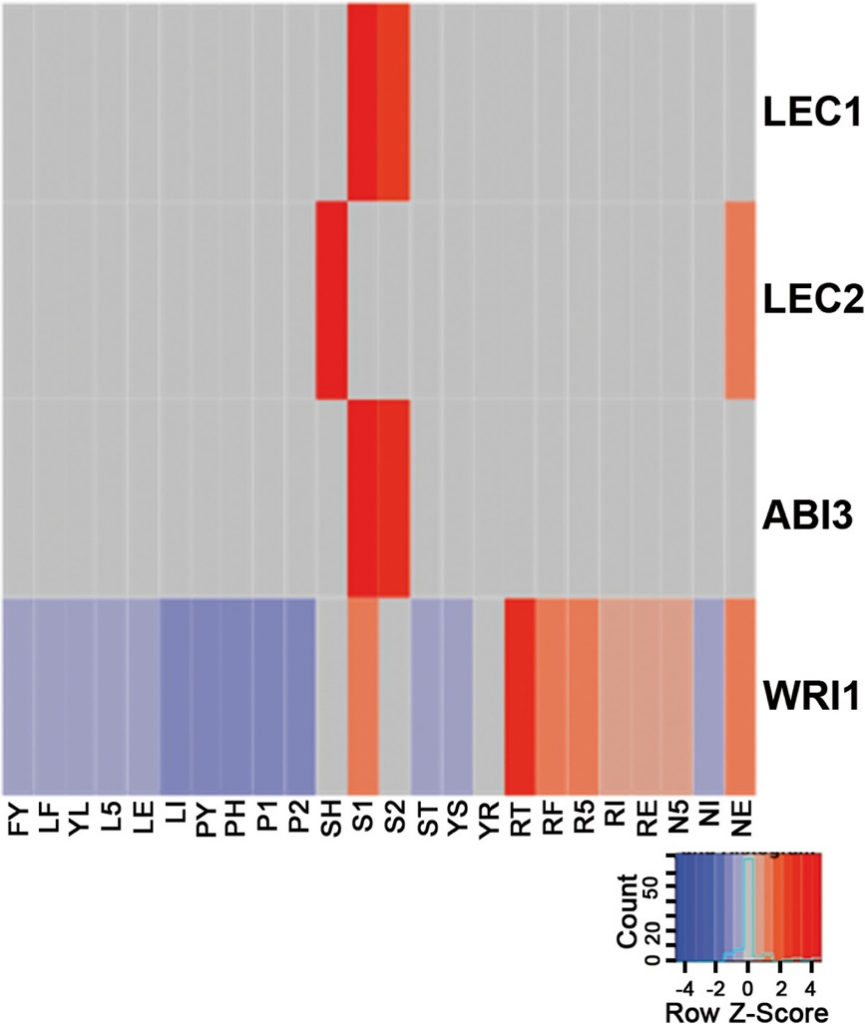
**Seed master transcription factor expression.** The expression profiles (as Z-scores: red = high, blue = low) of four transcription factors that regulate seed development in multiple species. Note the low expression of LEC2 (RPKM = 4) and WRI1 (RPKM = 5–9) in developing seeds. See Table 1 for tissue descriptions.

Seed development in multiple species is regulated by four master TFs: LEAFY COTYLEDON1 (LEC1), LEAFY COTYLEDON2 (LEC2), ABSCISIC ACID INSENSITIVE3 (ABI3), and WRINKLED 1 (WRI1*)* [21, 22, 30]. Using BLASTP, we queried the Arabidopsis protein sequences to identify homologous sequences in the *Pv* predicted genes (Figure 4). The homolog for *LEC2* was only weakly expressed (RPKM = 4) mid-seed development in *Pv*. Seeds of Arabidopsis loss of function *lec2* mutants accumulated 15% less protein and 30% less oil while the seed starch content increased five fold as compared to wild type plants [31]. The altered seed composition of *lec2* mutant plants closely resembles that of *Pv*, suggesting down regulation of *LEC2* may affect seed composition. *LEC2* controls the gene expression of WRI1, which also exhibits low expression patterns in *Pv* developing seeds (RPKM: SH = 5, S1 = 9, S2 = 5). *WRI1* expression modulates the expression of a set of genes controlling late glycolysis and fatty acid biosynthesis. The low expression of both *LEC2* and *WRI1* may relate to the lower oil composition of *Pv*.

Abscisic acid (ABA) is a key hormone in seed development, important in developing desiccation tolerance and entrance into dormancy [30]. ABA accumulation in seeds is both temporally and spatially regulated [27]. We found high expression of ABA biosynthesis genes (Phvul.002G018700.1 and Phvul.005G031500.1) in developing seeds, with expression decreasing as the seeds matured (Additional file 3g). ABA biosynthesis is regulated by 9-cis-EPOXYCAROTENOID DIOXYGENASE (NCED9) [32]. The expression pattern of *NCED9* (Additional file 3 h) and ABA biosynthesis genes (Additional file 3 g) in *Pv* developing seeds is consistent with those from developing seeds in Arabidopsis [33–35].

Trehalose biosynthesis is important in regulating both seed composition and nodule metabolism [36, 37]. In seeds, TREHALOSE 6 PHOSPHATE SYNTHASE 1 (TPS1), the enzyme responsible for converting glucose-6-phosphate to trehalose-6-phosphate is thought to regulate sugar utilization [32]. In *Arabidopsis thaliana tps1* null mutants, both sucrose and starch content of seeds dramatically increased [38]. In *Pv, TPS1* expression drops dramatically as the seed develops (Additional file 3i), corresponding with increased SS expression. We hypothesize that, as in Arabidopsis, the reduced *TPS1* expression promotes increased carbohydrate biosynthesis in *Pv* seeds.

### Nodule analysis

**Figure 5:**
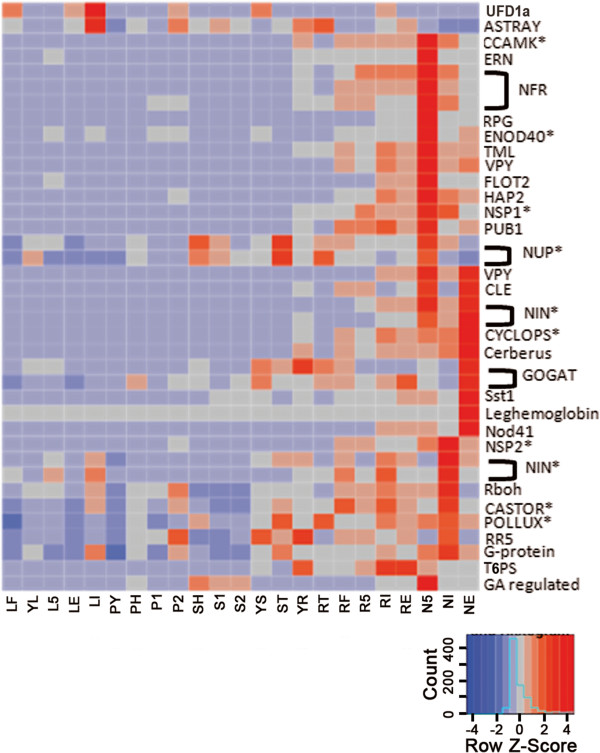
**Nodulation gene expression patterns.** Expression patterns (as Z-scores) of *Pv* homologs of genes involved in nodulation and symbiosis identified in *Lotus japonicus*, *Medicago truncatula*, and *Glycine max.* Red indicates a positive Z-score while blue indicates a negative Z-score. Genes common to both symbiotic nitrogen fixation and mycorrhizal symbiosis are denoted with an asterisk (*). See Table 1 for tissue descriptions.

Legumes have established a unique symbiotic relationship with *Rhizobium*, which allows legumes to fix atmospheric N_2_ into biologically useful NH_3_. For this experiment plants were provided with nutrients containing NO_3_^−^ nitrogen for optimal growth conditions or inoculated with either effective fix + *Rhizobium tropici* CIAT899 or ineffective fix- *Rhizobium giardini* 6917 to induce nodulation. Plants inoculated with normal (fix+) *R. tropici* appeared green and healthy, though smaller than plants provided with nitrate fertilizer (Additional file 13a). This phenotype is consistent with previous studies reporting the overall growth of N_2_ fixing *Pv* plants is restricted compared to fertilized plants, likely due to altered carbon partitioning [39, 40]. Fix + plants inoculated with *R. giardini* were nitrogen (N) deficient, exhibiting severe chlorosis and a stunted phenotype (Additional file 13a). Small, pre-fixing white nodules (N5) were isolated from root tissues of plants inoculated with effective fix + *R. tropici* five days after inoculation (DAI). At 21 DAI nodules were collected from plants inoculated with either fix + *R. tropici* (NE) or fix- *R. giardini* (NI) (Additional file 13b and c respectively). Microscopic imaging of fix-nodules 21 DAI (NI) revealed early senescing cells with few, if any, infected cells compared to fix + nodules formed 21 DAI (Additional file 13d and e). *In situ* hybridization analysis was used to visualize the localization pattern of leghemoglobin transcripts in these two nodule types. Fix- nodules collected 21 DAI (NI) exhibited little to no expression of leghemoglobin transcripts while fix + nodules collected 21 DAI (NE) exhibited high expression levels, likely mirroring the bacteroid colonization patterning (Additional file 13f and g) and directly reflecting the gene expression patterns observed in the RNA-Seq data (Figure 5). Nodule acetylene reduction assays failed to detect nitrogenase activity at 5 DAI in pre-fixing nodules and at 21 DAI with fix-nodules. Fix + nodules (and associated roots) from plants 21 DAI reduced 320 nm/hr/gfw (roots) of acetylene, indicating high N_2_ fixation activity. Leaf tissue was also collected at 5 DAI for fix + plants and 21 DAI for both fix + and fix- inoculated plants (see Table 1).

Comparing 5 DAI pre-fixing nodules (N5) and 21 DAI fix + nodules (NE) revealed 2,932 differentially expressed genes (Additional file 14). Comparing 21 DAI fix + and fix- nodules (NE vs NI) identified 2,953 differentially expressed genes (Additional file 15). Additionally, we found 245 nodule specific genes; genes expressed in any and/or all the nodule tissues sampled, but not expressed in any other tissues (Additional file 4). Comparing these nodule specific genes to those identified in two soybean gene atlases [26, 41] identified 21 nodule specific homologs common to both species (Additional file 16) including seven TFs and four transporters. The conserved expression of these genes highlights the importance of regulating gene expression, but also the exchange of nutrients between nodules and the plant roots. Five of these sequences have no known annotation, though nodule specific expression in both species suggests these are important candidates for characterization in future nodulation and nitrogen fixation research.

Cognate genes involved in nodule development and the establishment of N fixation have been identified and cloned from multiple species [42, 43]. Using BLASTN, the homologous sequences in common bean were identified and gene expression patterns were visualized as a heat-map (Figure 5). Upon further analysis of these nodule cognate genes, we detected three expression profiles: those highly up regulated early in nodule development (N5), those highly up regulated in 21DAI fix + nodules (NE), and those highly up regulated in 21 DAI fix- nodules (NI).

The autoregulation of nodulation (AON) pathway mediates nodule formation [44]. ASTRAY and UFD1a proteins, both expressed in leaves, function in the AON pathway [44]. *ASTRAY*, encodes a bZIP TF that interacts with a nodulation autoregulation receptor kinase (NARK) [44, 45]. *UFD1a* expression indicates the presence of Q, a root derived signal induced upon compatible *rhizobial* infection [44]. In soybean, three candidates for Q have been identified, all of which are CLE peptides [46]. *Pv* encodes a single CLE homolog (Phvul.005G097000.1), which is highly up regulated in N5 and NE, but noticeably absent in NI (Figure 5). Surprisingly, we observed aerial AON genes (*ASTRAY and UFD1a*) expressed higher in leaves of plants inoculated with fix- *rhizobia* than in leaves of plants inoculated with fix + rhizobia (Figure 5). We hypothesize the fix- inoculated plant may up regulate the AON pathway to minimize resources allocated to nodules as part of a survival strategy.

Early in nodule development (N5) nod factor receptors (NFR) and nodulation signaling pathway (NSP) TFs are highly expressed (Figure 5). The early calcium spiking response induces both a calmodulin dependent protein kinase (CCaMK/DMI3), which is required and sufficient for nodule organogenesis [47–49], and nuclear porin proteins (NUPs) [44] (Figure 5). Additionally, genes involved in infection thread formation and elongation including *ERN1, FLOT, VPY, PUB1* and *RPG* [50–58] are highly up regulated in N5 (Figure 5). *VPY and PUB1*, which are up regulated in N5 and NE, are involved in *rhizobial* recognition, attachment, entry, and initiation of the infection thread [53, 58]. Nodule organogenesis involves the altered differentiation and division of root cortical cells prior to the formation of the nodule primordia. In *Medicago truncatula*, these processes are dependent on *ENOD40* [59], which is highly expressed in N5 (Figure 5). *HAP2*, which promotes nodule development and the release of bacteria from the infection thread [60] is expressed highest in N5, but remains elevated in NE (Figure 5).

Genes highly expressed in NE are involved in processes such as amino acid biosynthesis, nitrogen metabolism, carbohydrate metabolism, membrane transport, and sulfur assimilation (cysteamine dioxygenase). We identified 402 genes highly expressed in NE as compared to all other tissues (Additional file 17 and Additional file 18). These genes are likely involved in the establishment of symbiosis and symbiotic nitrogen fixation (SNF). Among these 402 genes, 73 encoding a transmembrane domain, 49% of which relate to transport including Phvul.002G300900.1, which encodes a SWEET4 homolog. SWEET genes mediate sucrose transport to the phloem [61]. In Arabidopsis, SWEET4 is up regulated by pathogen infection, likely being co-opted to aid pathogen growth [62]. We hypothesize this function is conserved in *Pv* upon fix + *Rhizobum* infection. Consistent with these results, the two most statistically significant GO categories among the 402 genes are GO:0005215 (P-value = 0.002), associated with transport activity and GO:0006857, associated with oligopeptide transport (P-value = 0.007). Increased expression of transporters in nodule tissues is consistent with previous reports [63] of nodule organogenesis gene expression. Also important to nodule function is carbohydrate metabolism, which is statistically over-represented in genes highly expressed in NE (Table 3, GO:0030246) [63]. Among the 402 genes up regulated in NE are 34 TFs belonging to 18 different families and 73 transporters. Four of the TFs belong to the Nodule Inception (NIN) family. NIN TFs mediate signals of rhizobial infection including: root hair curling, infection thread formation, and the initiation of the nodule primodia [64–66]. They are also involved in regulating gene expression in response to nitrate. Additional highly expressed genes in NE are members of the shi related sequence (SRS) TF family, with 32% of the familial expression from nodules (Figure 1). SRS TFs mediate protein:protein interactions involved in ubiquitination for targeted proteolysis. Expression data from both *Pv* and soybean [26, 41] indicates this family is highly expressed in both roots and nodules of legumes (Figure 1).

**Table 3:** **Gene Ontology (GO) categories statistically over-represented in NE enhanced genes**

GO ID	P-value	Number of genes ^a^	Description
GO:0005215	0.00271	14	Transporter Activity
GO:0006857	0.00702	8	Oligopeptide Transport
GO:0004106	0.00871	3	Chorismate Mutase Activity
GO:0006188	0.01178	2	IMP Biosynthetic Process
GO:0006950	0.01773	7	Response to Stress
GO:0047800	0.01929	3	Cysteamine Dioxygenase Activity
GO:0030246	0.01929	3	Carbohydrate Binding
GO:0009073	0.01929	3	Aromatic Amino Acid Biosynthesis
GO:0006164	0.02765	2	Purine Nucleotide Biosynthesis
GO:0009113	0.02765	2	Purine Base Biosynthesis
GO:0030259	0.03735	3	Lipid Glycosylation

^a^The number of genes on NE enhanced list with GO ID of interest.

Among the nodule cognate genes, those most highly expressed in NE are: NIN transcription factors, *CYCLOPS*, and *CERBERUS* with expression profiles increasing from 5 DAI to 21 DAI in fix + nodules (2X, 2.4X, and 4X respectively, Figure 5). *CYCLOPS* expression is required for rhizobia infection. Nodule organogenesis is dependent on *CERBERUS* gene expression [49, 67–69].

The primary function of nodules is to fix N_2_ to NH_3_. Nitrogenase, the enzyme responsible for nitrogen fixation, requires sulfur and a near anaerobic environment to function. The gene encoding SYMBIOSOME SULFATE TRANSPORTER 1 (SST1), which transports sulfur into bacteroids [70], is expressed 15 fold higher in NE than in NI (Figure 5). The genes encoding leghemoglobin, which sequesters oxygen [71], are only expressed in NE (Figure 5). Once N_2_ is reduced to usable ammonia it must be assimilated for use and distribution throughout the plant. NADH-dependent glutamate synthase (NADH-GOGAT) is a key enzyme in ammonia assimilation [72]. Two NADH-GOGAT genes (Phvul.009G053900.1 and Phvul.001G076400.1) are expressed 5 and 10-fold higher in NE than in NI (Figure 5), reflecting the difference in effectiveness. Transcripts encoding glutamine synthase, uricase, and allantoinase (Additional file 3 j, c, and d), each involved in primary ammonia assimilation, exhibit similar expression patterns. Nod41 was identified in *Pv* by Olivares *et al*. [73] as important in controlling defense responses during symbiotic interactions and maintaining the integrity of the uninfected root nodule cells. Our data is consistent with this hypothesis as *Nod41* is expressed 7-fold higher in 21 DAI fix + nodules than in 21 DAI fix- nodules (Figure 5).

Genes up regulated in NI include those involved in the GO processes of autophagy and early senescence including ubiquitination, proteolysis, peptidyolysis and apoptosis. Respiratory burst oxidase homolog (RBOH) genes, which generate reactive oxygen species (ROS) [74], are up regulated four-fold in NI. Increased ROS production is a common defense response to pathogen attack (ie: ineffective rhizobia) and in response to abiotic stress, including nitrogen deficiency. Additionally, we observed high expression of leucine rich repeat (LRR) genes in NI (Additional file 19), likely reflecting a defense response as the plant reacts to invading bacteria [75]. Also highly expressed in NI are genes involved in oxidation-reduction processes, membrane transport, protein binding, and amino acid phosphorylation. Among the nodule cognate genes are those encoding a second group of NIN and NSP TFs (Figure 5). This result suggests a group of alternative TFs may be induced in NI versus NE.

Type A-response regulators (RRs) negatively regulate cytokinin signaling [76]. In lotus, RRs are rapidly induced following *rhizobial* inoculation in root hairs and dividing cortical cells [77], repressing the cytokinin signaling pathway [48]. Inhibition of the cytokinin-signaling pathway may contribute to plant and bacterial cell differentiation. In *Pv*, the gene encoding RR5 is more highly expressed in NI than in NE (Figure 5). Genes encoding both CASTOR and POLLUX, both of which are required for the activation of voltage gated calcium (Ca^2+^) channels [74], are highly expressed in NI (Figure 5). The high expression of *CASTOR* and *POLLUX* genes in NI may suggest that at 21 DAI the plant is still attempting to establish SNF or may reflect the induction of Ca^2+^ channels by ROS as described in *Pisum sativum* [78, 79].

### Roots and nitrogen

Gene expression profiles of *Pv* roots were examined from plants grown under three conditions 1) those from NO_3_^−^ fertilized plants (RF), 2) those from plants with fix + effective nodules (RE), and 3) those derived from plants having fix- ineffective nodules (RI). RF and RE roots had adequate N for growth, while RI roots were N deficient. We identified 1,714 genes differentially expressed between the three 21 DAI root samples (RI, RE, and RF) (Additional file 20). The majority of these genes (1,668 genes) are differentially expressed between fertilized roots (RF) and nodulated roots (either RE or RI).

**Figure 6:**
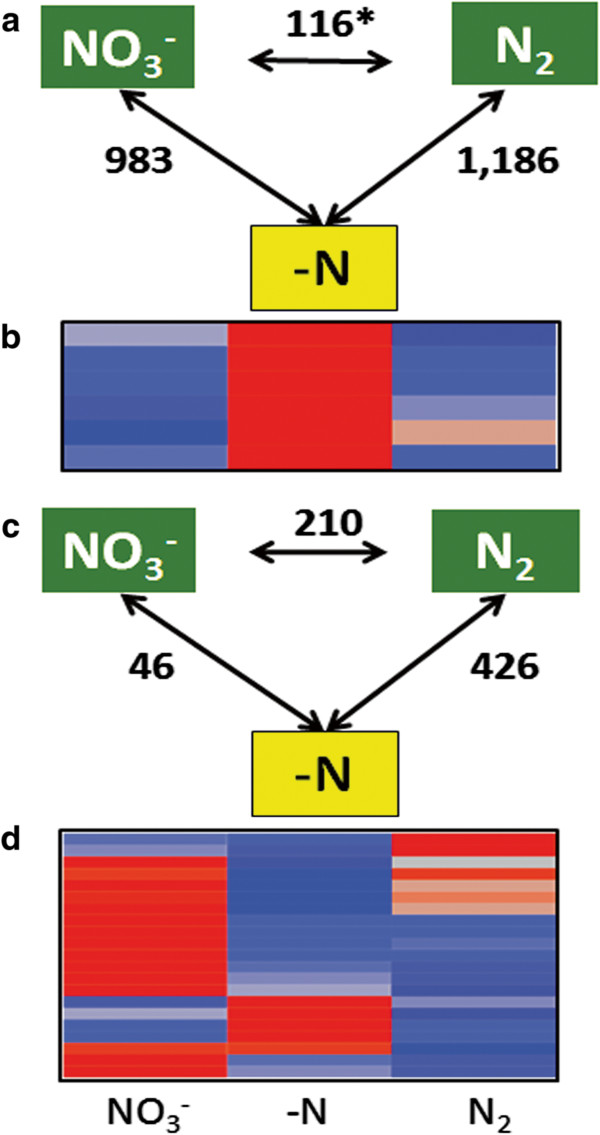
**Impact of nitrogen source on gene expression patterns.** Genes differentially expressed between leaf samples **(a)** and root samples **(c)** due to the nitrogen source. Heatmaps of gene expression profiles, represented by Z-scores; red indicates a positive Z-score, blue indicates a negative Z-score. **(b)** auxin response factor expression in leaves. **(d)** nitrogen transporter expression; plants provided with NO_3_^−^ up regulate low affinity N transporters 1 and 3, while N deficient plants up regulate high affinity transporter NRT2. Plants fixing N_2_, show an increased expression of NRT1.

Comparing gene expression patterns between RI (roots from fix- plants) and both RF and RE identified 426 and 46 genes differentially expressed between root samples respectively (Figure 6c). Additionally, 210 genes were differentially expressed between RE and RF. These 210 sequences represent genes differentially expressed due to the N source (Figure 6c). A similar comparison of leaf tissues collected from each of the plants revealed 116 genes differentially expressed between + N leaves due to different N sources (Figure 6a). These 116 genes indicate that the source of N (either via N_2_ fixation or NO_3_^−^ fertilization) has a long-term impact on plant gene expression. Among all 2,641 genes differentially expressed between samples due to the N source are 340 TFs, the majority of which are up regulated in –N tissues (Additional file 21 a and b).

In the presence of abundant NO_3_^−^, plants will preferentially take up and utilize NO_3_^−^ rather than develop SNF. NO_3_^−^ transporters exhibit either low (NRT1) or high (NRT2 and NRT3) NO_3_^−^ affinity [80–84]. Examination of the expression patterns of NO_3_^−^ transporters in our root samples revealed plants provided with NO_3_^−^ as a fertilizer induce *NRT1* and *NRT3* gene expression, reflecting the abundance of available NO_3_^−^ (Figure 6d). In –N roots, only members of the high affinity NRT2 gene family are up regulated (Figure 6d). N-deficient plants may up regulate N transporters in an attempt to increase the N content of the plant to mitigate –N stress. Members of the low affinity NRT1 gene family are also up regulated in the roots of N_2_ fixing plants (Figure 6d). The constitutive expression of *NRT1.1* is consistent with the recent evidence suggesting that it serves as both an N sensor and transporter [85–87]. Expression of *NRT1.1* in fix + plants may be involved in N sensing.

Once N is within the plant it must be assimilated. Glutamine synthetase (GS) functions as a primary enzyme for NH_4_ assimilation produced from N_2_ fixation or NO_3_^−^ nutrition [88, 89]; synthesizing glutamine from NH_3_ and glutamate (Additional file 3j). In SNF plants, the majority of glutamine is committed to the *de-novo* purine biosynthesis pathway. Alternatively, glutamine may be reduced by GOGAT. Consistent with previous studies [72], *NADH-GOGAT* expression is highest in roots (particularly YR) and NE while *Fd-GOGAT* is expressed highest in leaf tissues (Additional file 3 k). Plants provided with NO_3_^−^ fertilization utilize glutamine in the synthesis of asparagine via asparagine synthetase (AS) [88], which is most highly expressed in fertilized root (RF) tissues (Additional file 3 l).

Our data shows increased expression of auxin response factors unique to –N leaves (Figure 6b). This gene expression pattern indicates increased auxin levels in –N leaves, supporting auxin as the N signal. The availability of N for proper growth and development is likely monitored throughout the plant. Auxin has been proposed as an N status mediator, signaling from root to shoot [80]. Under low N and other nutrient stress conditions in the shoot, increased auxin is transported to the roots to enhance lateral root development, a hallmark response of –N plants [46, 80, 87].

## Conclusion

This study provides a resource for global analysis of gene expression patterns in *Pv* of 24 samples from seven unique tissues across important developmental time points. The publicly available gene atlas, *Pv* GEA, will facilitate the use of this data for researchers querying gene expression patterns within various biological processes, as evidenced by Additional file 3. Additionally, by comparing gene expression patterns in developing seeds of *Pv* to those in *Glycine max*, we were able to identify differences potentially responsible for altered seed composition between the two closely related species. Finally, our analysis of N uptake and utilization revealed the N source is an important component of the N pathway and has a long-term effect on gene expression patterns.

## Methods

### Plant materials and growth conditions

*Phaseolus vulgaris* cv. Negro jamapa seeds were grown as described by O’Rourke et al. [90]. At the emergence of the unifoliate, pots were assigned to one of three nitrogen (N) treatments; inoculated with *Rhizobium tropici* CIAT899 (fix+), *Rhizobium giardini* 6917 (fix-), or fertilized with a full nutrient solution. Pots assigned to the fertilization treatment were watered daily with a nutrient solution of 9 mM KNO_3_, 2.5 mM Ca(NO_3_)_2_, 1.0 mM Ca(H_2_PO_4_)_2_, 1.0 mM MgSO_4_, 12 μM Fe (as FeEDTA), 4.0 μM MnCl_2_, 22.0 μM H_3_BO_3_, 0.4 μM NaMoO_4_, and 1.6 μM CuSO_4_. Twenty-four tissue samples were collected throughout development and across all N treatments (for details see Table 1). For each nitrogen treatment, a representative plant was chosen and leaf, root, and nodule tissue samples were collected. Two plants were maintained in full nutrient solution fertilized pots. From these plants, root, leaf, flower, stem, seed and pod tissues were collected (see Table 1 for details). All tissue collected for RNA -Seq analysis was immediately flash frozen in liquid nitrogen.

### RNA extraction and expression analysis

Total RNA was purified from 24 tissue samples using RNeasy Plant Mini Kit (Qiagen, Valencia, CA, USA). For RNA-Seq analysis, RNA samples were shipped on dry ice overnight to National Center for Genome Resources (NCGR, Santa Fe, NM) for sequencing as described by Severin et al. [26]. Illumina reads generated from all 24 samples are available at the NCBI SRA browser, accession number SRP046307. Illumina reads passing quality control standards, approximately 25 million sequences per sample or 596 million 36 bp sequences total, were mapped to the *Phaseolus vulgaris* v1.0 genome available at http://www.phytozome.net using the program Bowtie [91]. Reads were also mapped to the predicted transcripts to account for splicing events. Reads that mapped to more than one location were counted at each mapping location. Of the 596 million reads generated, 406 million (89%) mapped to the genome with 14% of those mapping to non-coding regions. Raw gene expression counts were normalized using the RPKM (reads/Kb/Million) method [92, 93] using custom R scripts. To ensure expression profiles were not statistical artifacts as described by earlier studies [94, 95], we determined an RPKM of 3 represents a 2X coverage across the coding region, assuming equal distribution, and would be the minimum level at which a gene would be considered expressed; genes with an RPKM < 3 were considered silent. Transcripts differentially expressed between libraries were identified using NOIseq [96]. Differentially expressed transcripts were required to have > 2-fold change in expression between samples and a probability of differential expression > 0.9. Additionally, one of the two sequences was required to have an RPKM > 3. Heatmaps illustrating expression patterns of various subgroups of transcripts were generated in R as described by Severin *et al.* [26].

To identify genes exhibiting enhanced expression in NE, we determined the Euclidian distance between Z-scores for each gene. A threshold of two Euclidian distances was established as significant; genes within the threshold were identified as co-expressed.

### Real time quantitative RT-PCR (qPCR) and housekeeping genes

RNA was extracted using the RNeasy Plant Mini Kit (Qiagen, Valencia, CA, USA) from three biological replicates of tissues grown in growth chambers under the same conditions described above. Transcript specific primers were designed using Primer3 (Frodo.wi.mit.edu). The qPCR analysis was run as described by O’Rourke et al. [90] for 85 genes identified as differentially expressed by NOIseq (Additional file 6). 92% of the qPCR experiments confirmed the differential expression measured by NOIseq analysis in at least two of the three biological replicates.

Genes exhibiting stable expression profiles between tissues and across growth conditions were identified as described by Severin et al. [26] (Additional file 22). The 10% of transcripts with the lowest CV were selected as potential housekeeping genes. This suite of stably expressed transcripts may be useful in future experiments for normalizing gene expression patterns across a variety of experimental conditions, or tissues [97]. One of these genes (Phvul.006G165300.1) was successfully utilized as a housekeeping sequence in the qPCR analysis. Comparing the housekeeping genes proposed in this study to the eleven potential housekeeping genes identified for *Pv* under biotic and abiotic stress by Borges et al. [98] found seven genes common to both lists, illustrating the utility of this list for multiple experimental conditions.

### Acetylene reductase assay

Acetylene reduction assays were performed as described by Vance et al. [99] with the following modifications. Plant roots from six biological replicates of each sample (roots inoculated with fix + *R. tropici* CIAT899 at 5 and 21 DAI and roots inoculated with fix – *R. giardini* 6917 21 DAI) were placed in 500 ml airtight glass containers equipped with serum stoppers. 50 ml of air was removed from each container and replaced with 50 ml of ethylene; samples were incubated at room temperature for one hour, at which time 10 ml of gas was withdrawn from the container for analyses as previously described by Vance et al. [99].

### Nodule *In Situs*

A partial coding sequence for *Pv* leghemoglobin (645 bp) was PCR amplified and cloned into pBSSK+. Nodules inoculated with fix + *R. tropici* CIAT899 and fix – *R. giardini* 6917 were collected 21 DAI and analyzed as described by Sbabou et al. [100].

### Availability of supporting data

The expression data used in this study is publicly available at the NCBI short read archive; accession SRP046307. Additionally, the raw and normalized datasets can be downloaded and explored at the *Phaseolus vulgaris* Gene Expression Atlas (*Pv* GEA) website, http://plantgrn.noble.org/PvGEA/.

## Electronic supplementary material

Additional file 1: **Pairwise identification of differentially expressed genes.** (XLSX 76 KB)

Additional file 2: **Tissue specific genes.** (XLSX 172 KB)

Additional file 3: **Expression patterns of specific genes of interest.** Graphs illustrating the expression patterns of genes of interest, individual tissues on the X-axis, RPKM values, on the Y-axis. (a) tissue specific, (b) sample specific genes, (c) uricase (d) allantoin degradation, (e) glutamate dehydrogenase, (f) transcription factors with increased expression as seeds develop, (g) ABA1 and ABA2, (h) NCED9, (i) trehalose 6 phosphate, (j) glutamine synthetase, (k) GOGAT, (l) asparagine synthetase. (PDF 1 MB)

Additional file 4: **Sample specific genes.** (XLSX 207 KB)

Additional file 5: **Purine Biosynthesis.** Expression patterns of the *Pv* homologs in the purine biosynthesis pathway. Genes in this pathway are most highly expressed in effective nodules compared to the rest of the plant. For tissue descriptions see Table 1. Gene expression is represented by Z-scores; red indicates a positive Z-score while blue indicates a negative Z-score. (TIFF 3 MB)

Additional file 6: **qPCR results.** (XLSX 65 KB)

Additional file 7: **Transcription factors.** (XLSX 514 KB)

Additional file 8: **Genes differentially expressed between SH and PH.** (XLSX 592 KB)

Additional file 9: **Genes differentially expressed between S1 and P1.** (XLSX 823 KB)

Additional file 10: **Genes differentially expressed between S2 and P2.** (XLSX 925 KB)

Additional file 11: **Genes differentially expressed as seeds develop.** (XLSX 230 KB)

Additional file 12: **Genes differentially expressed as pods develop.** (XLSX 1 MB)

Additional file 13: **Nitrogen Deficiency, Plants, Nodules, and** ***In situs.*** (a) Plant phenotypes, plant on the left provided with nitrate (NO_3_^−^) fertilizer, middle plant inoculated with fix + *rhizobium*, plant on right inoculated with fix- *rhizobium* (nodules form, but no N_2_ fixation). (b) Effective (fix+) nodules 21 DAI with fix + *R. tropici* CIAT 899. (c) Ineffective (fix-) nodules 21 DAI with fix- *R. giardini 6917.* (d and e) Cross secti*on of fix +* (d) and fix- (e) nodules stained with toluene blue. Bacteroid structures visible in fix + nodules. Disorganized cellular structure evident in (e). (f and g) Transcript abundance of leghemoglobin: high in fix + nodules (f) but barely detectable in fix- nodules (g), as assessed by *in situ* hybridizations. (TIFF 8 MB)

Additional file 14: **Genes differentially expressed between NE and N5.** (XLSX 603 KB)

Additional file 15: **Genes differentially expressed between NE and NI.** (XLSX 555 KB)

Additional file 16: **Nodule specific genes common to** ***Pv*** **and** ***Glycine max.*** (XLSX 61 KB)

Additional file 17: **Genes highly expressed in NE compared to other tissues.** (XLSX 162 KB)

Additional file 18: **Genes highly up regulated in NE.** (TIFF 3 MB)

Additional file 19: **LRR gene expression.** 151 genes containing an LRR domain expressed in our data. Expression, represented by Z-scores, of LRR containing sequences is low in developing seeds, but high in ineffective nodules. Red indicates a positive Z-score while blue indicates a negative Z-score. For tissue description see Table 1. (TIFF 4 MB)

Additional file 20: **Genes differentially expressed between root samples.** (XLSX 183 KB)

Additional file 21: **Impact of N on TF expression.** Transcription factors differentially expressed in leaf samples (a) and root samples (b) of plants provided with NO_3_^−^ fertilizer, −N plants (inoculated with fix- rhizobium), and N_2_ fixing plants (inoculated with fix + rhizobium). Heatmaps of gene expression represented by Z-scores; red indicates a positive Z-score while blue indicates a negative Z-score. (TIFF 1 MB)

Additional file 22: **Housekeeping genes.** (XLSX 693 KB)
