# Supplementary figures and images for: An RNA-Seq based gene expression atlas of the common bean
Source: BMC Genomics. 2014 Oct 6;15(1):866. doi: 10.1186/1471-2164-15-866 (PMC4195886; doi:10.1186/1471-2164-15-866)

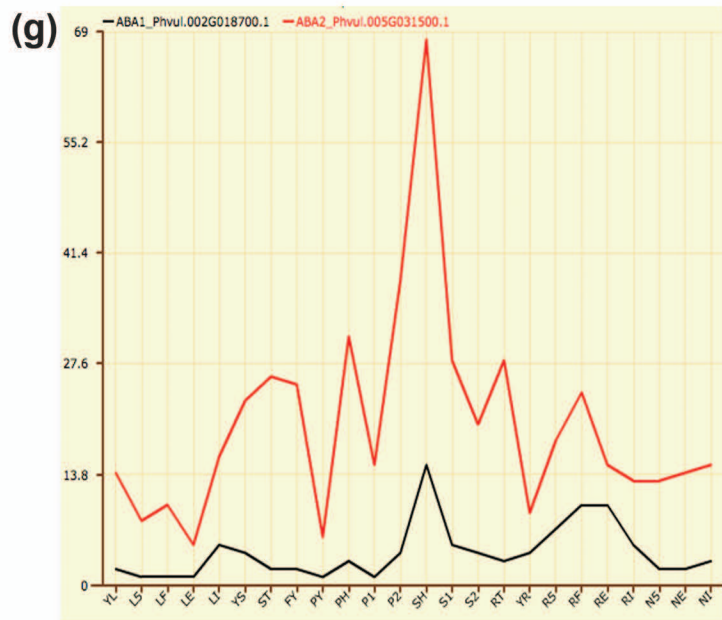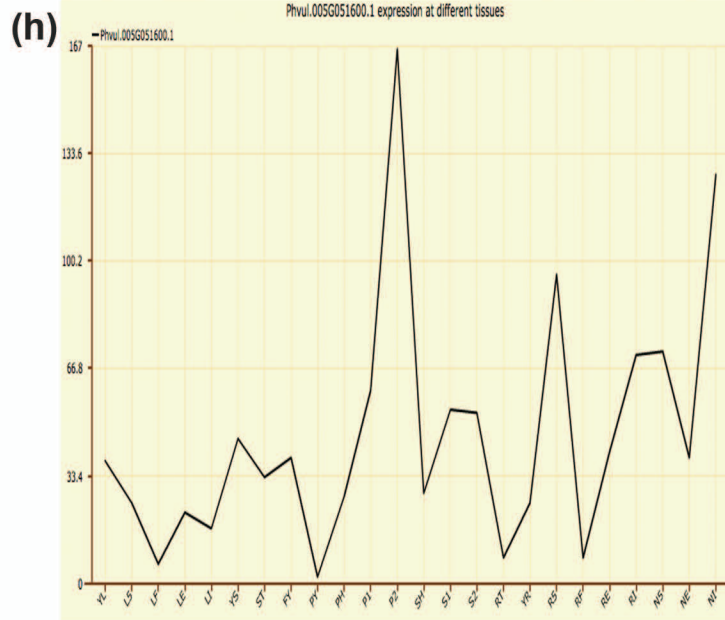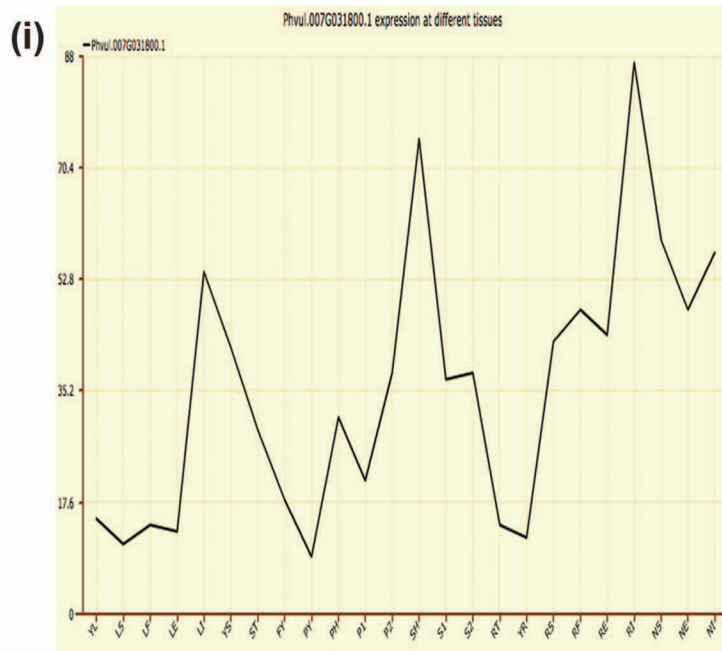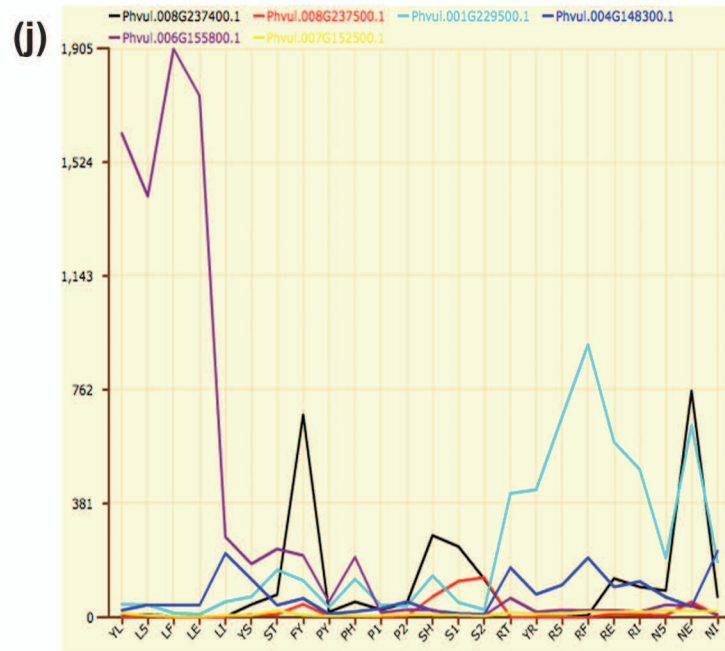

(k)

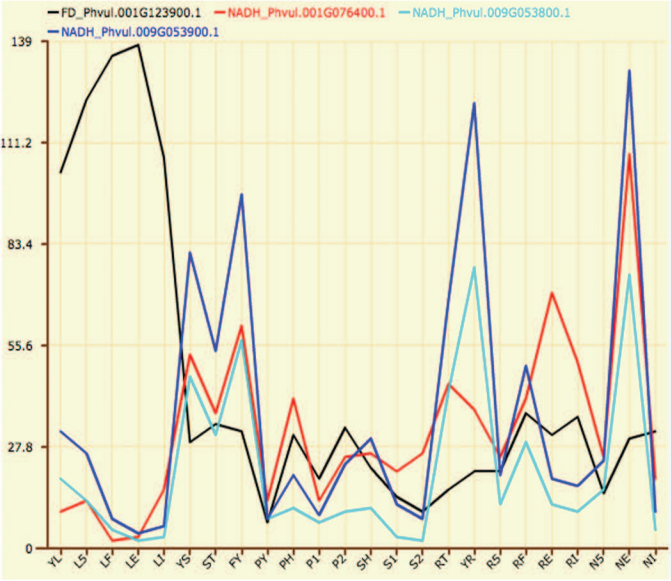

(l)

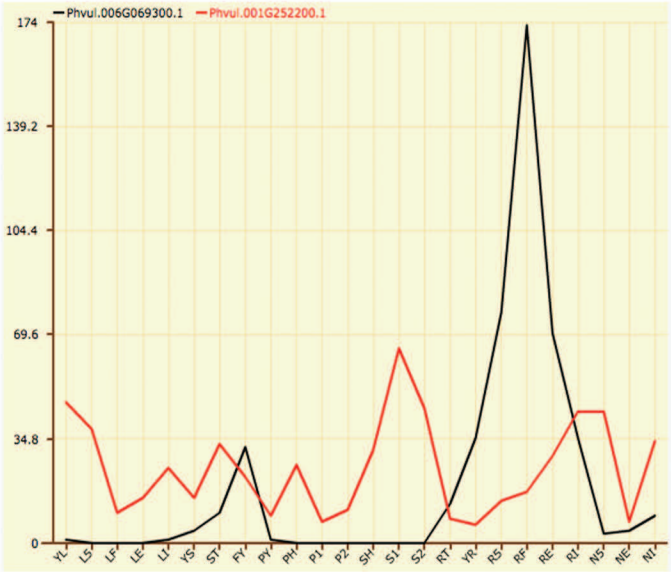

Supplement: Supplementary file 3 — Additional file 3: Expression patterns of specific genes of interest. Graphs illustrating the expression patterns of genes of interest, individual tissues on the X-axis, RPKM values, on the Y-axis. (a) tissue specific, (b) sample specific genes, (c) uricase (d) allantoin degradation, (e) glutamate dehydrogenase, (f) transcription factors with increased expression as seeds develop, (g) ABA1 and ABA2, (h) NCED9, (i) trehalose 6 phosphate, (j) glutamine synthetase, (k) GOGAT, (l) asparagine synthetase. (PDF 1 MB) [file 12864_2014_6531_MOESM3_ESM.pdf]

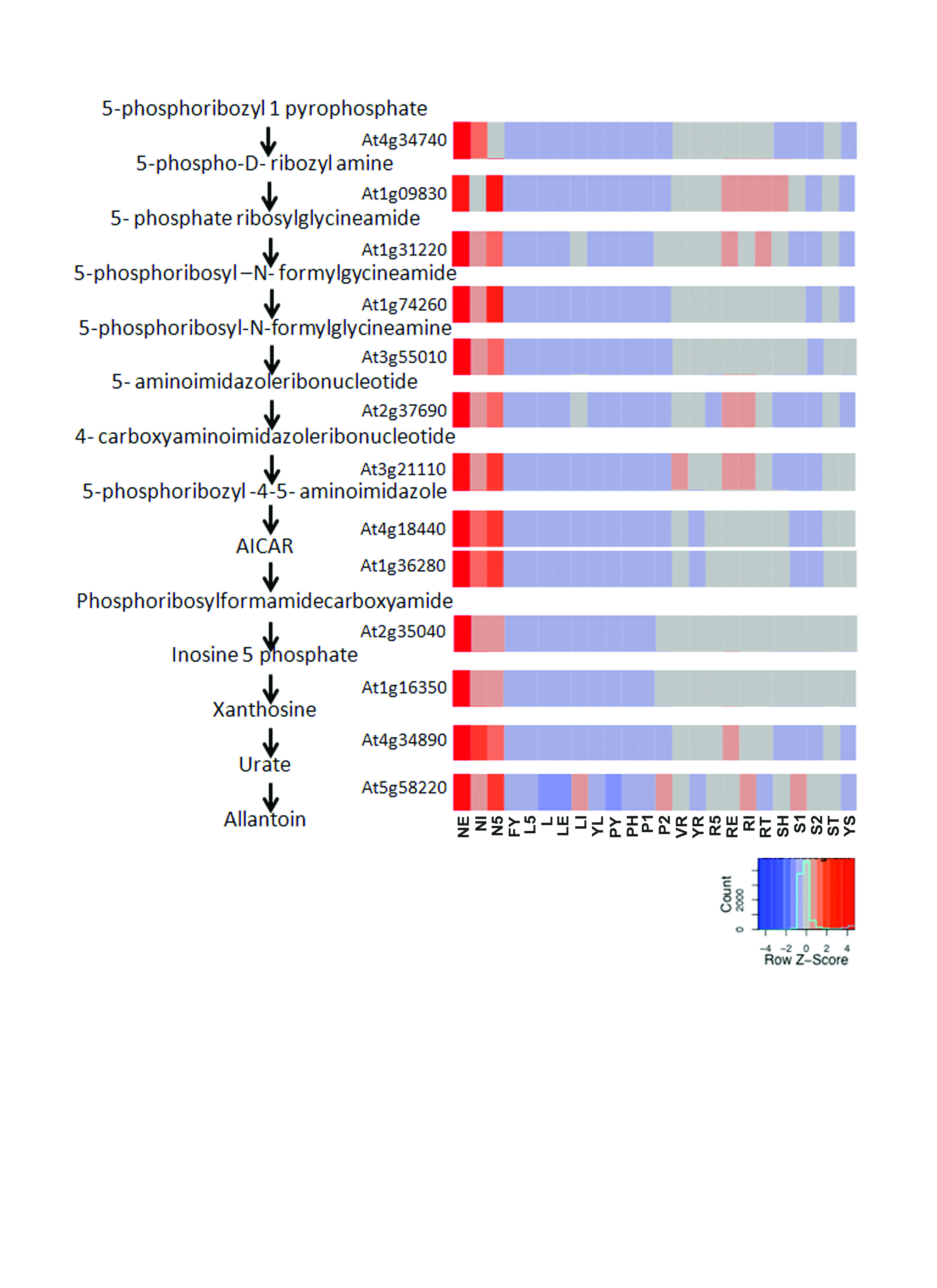

Supplement: Supplementary file 5 — Additional file 5: Purine Biosynthesis. Expression patterns of the Pv homologs in the purine biosynthesis pathway. Genes in this pathway are most highly expressed in effective nodules compared to the rest of the plant. For tissue descriptions see Table 1. Gene expression is represented by Z-scores; red indicates a positive Z-score while blue indicates a negative Z-score. (TIFF 3 MB) [file 12864_2014_6531_MOESM5_ESM.tiff]

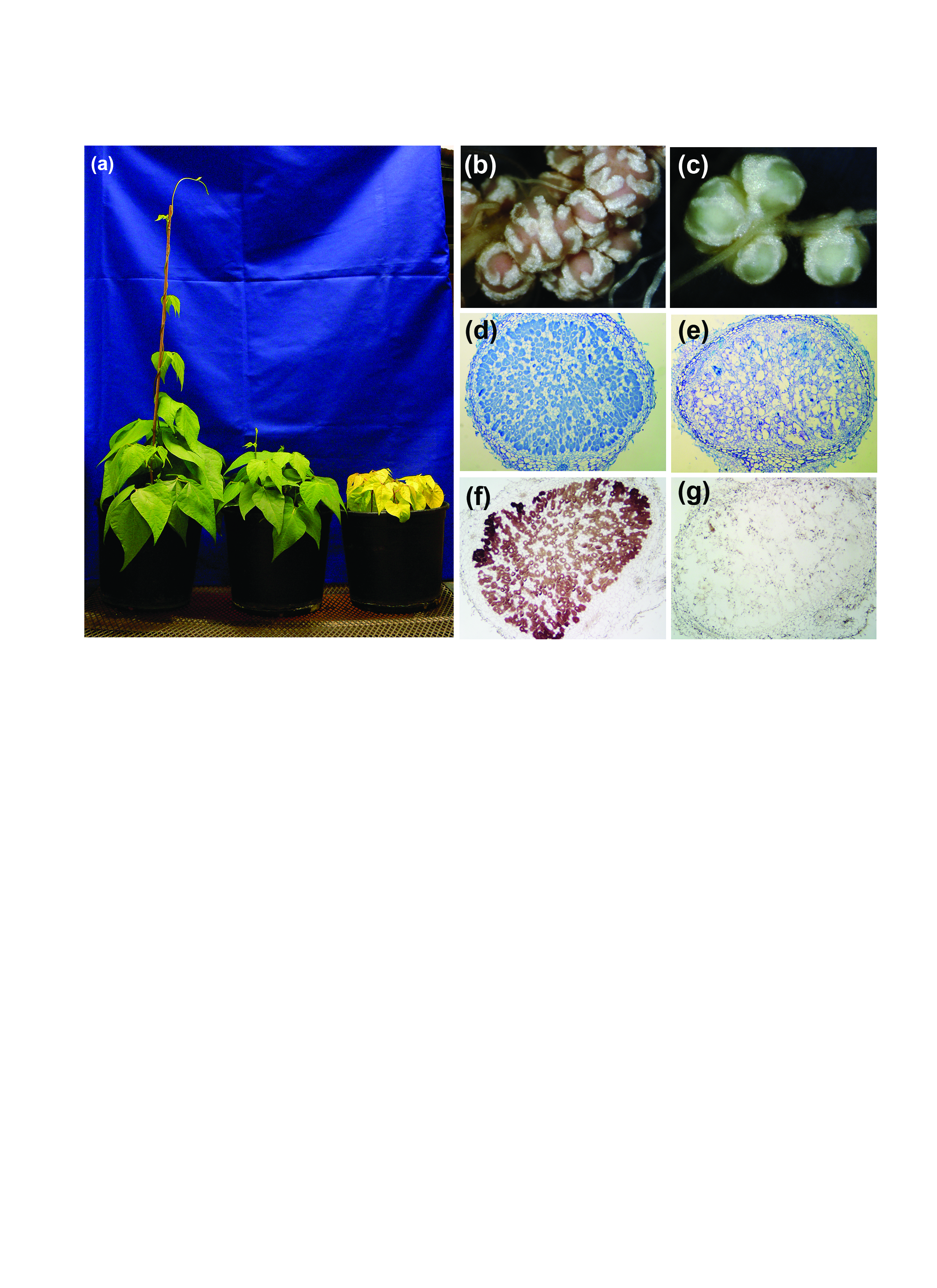

Supplement: Supplementary file 13 — Additional file 13: Nitrogen Deficiency, Plants, Nodules, and In situs. (a) Plant phenotypes, plant on the left provided with nitrate (NO3 −) fertilizer, middle plant inoculated with fix + rhizobium, plant on right inoculated with fix- rhizobium (nodules form, but no N2 fixation). (b) Effective (fix+) nodules 21 DAI with fix + R. tropici CIAT 899. (c) Ineffective (fix-) nodules 21 DAI with fix- R. giardini 6917. (d and e) Cross section of fix + (d) and fix- (e) nodules stained with toluene blue. Bacteroid structures visible in fix + nodules. Disorganized cellular structure evident in (e). (f and g) Transcript abundance of leghemoglobin: high in fix + nodules (f) but barely detectable in fix- nodules (g), as assessed by in situ hybridizations. (TIFF 8 MB) [file 12864_2014_6531_MOESM13_ESM.tiff]

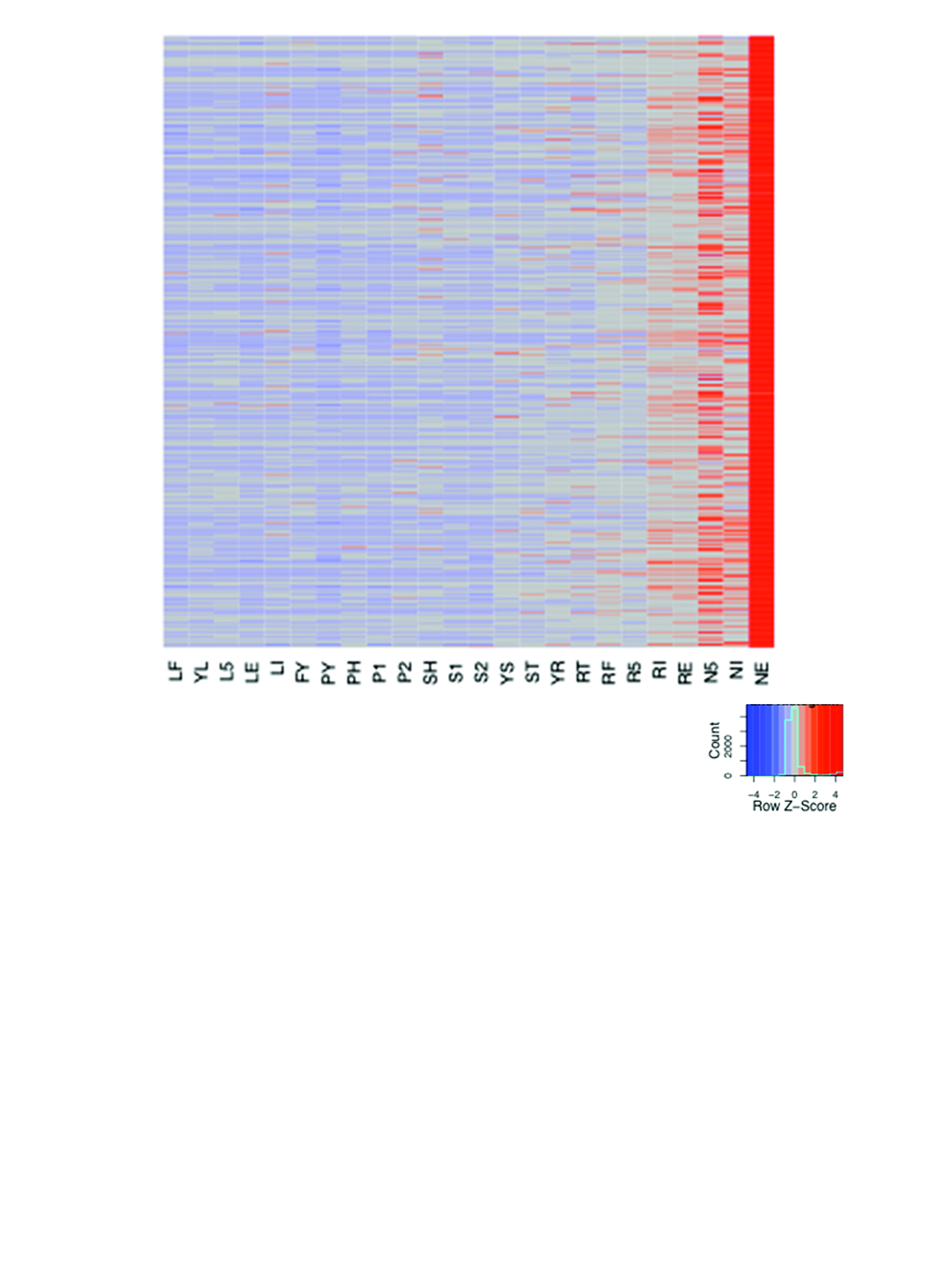

Supplement: Supplementary file 18 — Additional file 18: Genes highly up regulated in NE. (TIFF 3 MB) [file 12864_2014_6531_MOESM18_ESM.tiff]

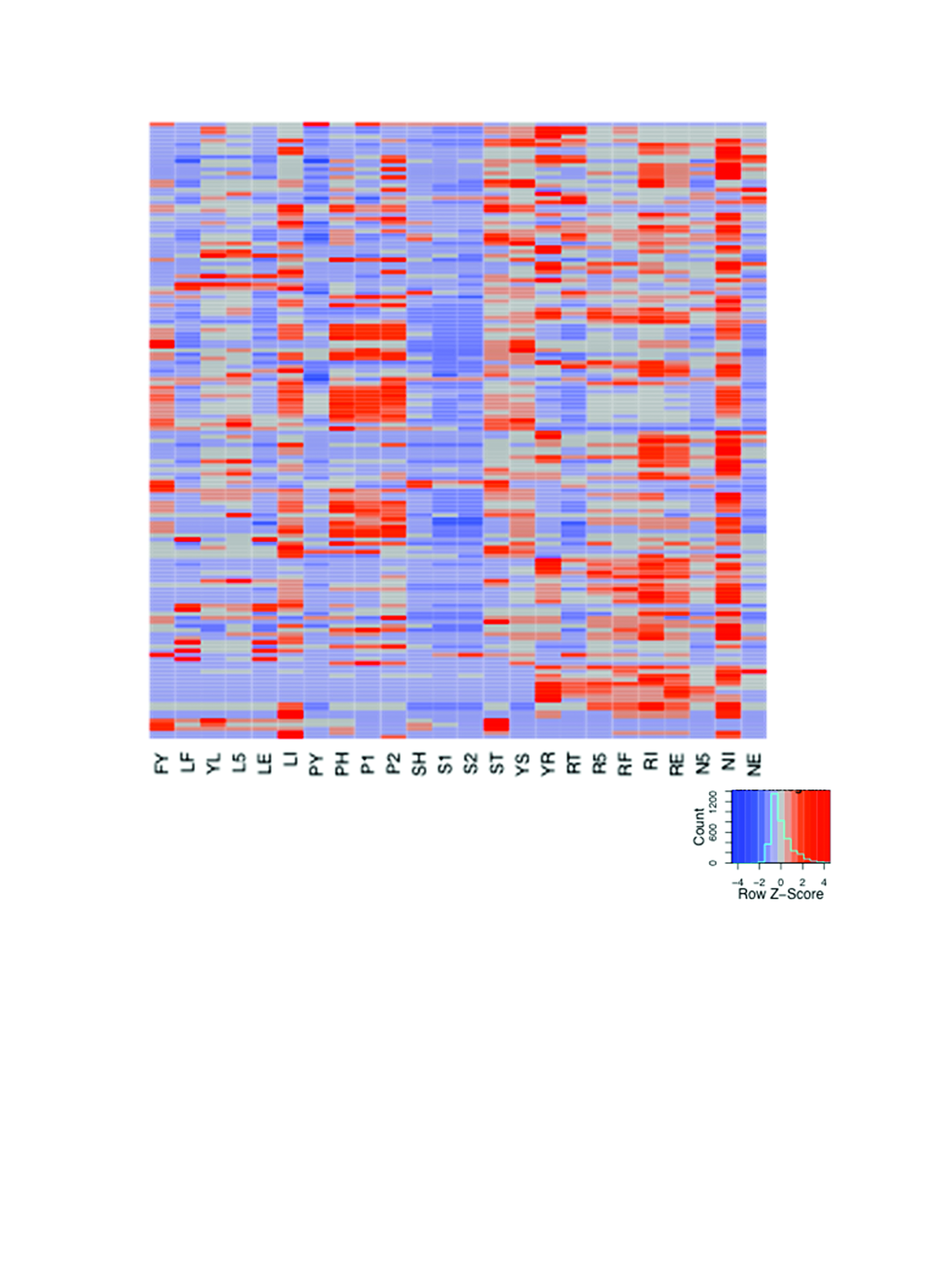

Supplement: Supplementary file 19 — Additional file 19: LRR gene expression. 151 genes containing an LRR domain expressed in our data. Expression, represented by Z-scores, of LRR containing sequences is low in developing seeds, but high in ineffective nodules. Red indicates a positive Z-score while blue indicates a negative Z-score. For tissue description see Table 1. (TIFF 4 MB) [file 12864_2014_6531_MOESM19_ESM.tiff]

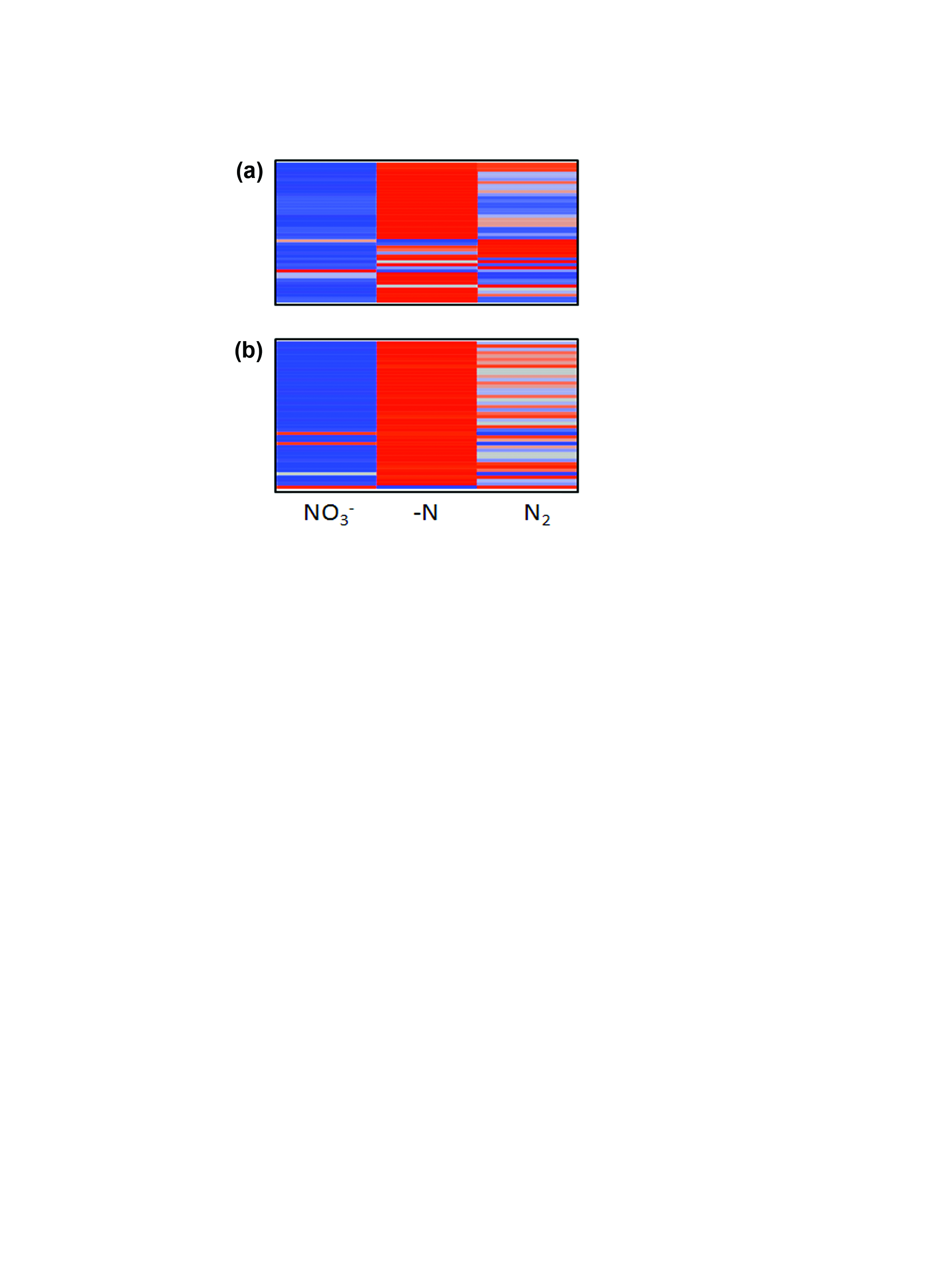

Supplement: Supplementary file 21 — Additional file 21: Impact of N on TF expression. Transcription factors differentially expressed in leaf samples (a) and root samples (b) of plants provided with NO3 − fertilizer, −N plants (inoculated with fix- rhizobium), and N2 fixing plants (inoculated with fix + rhizobium). Heatmaps of gene expression represented by Z-scores; red indicates a positive Z-score while blue indicates a negative Z-score. (TIFF 1 MB) [file 12864_2014_6531_MOESM21_ESM.tiff]
